# Athletes and Medical Staff in Elite Contact Sports: a Quantitative Analysis of Use, Knowledge, and Attitudes Regarding Cannabinoids

**DOI:** 10.1177/19417381261495512

**Published:** 2026-10-21

**Authors:** Elizabeth S. Thompson, Jane Alcorn, Robert Laprairie, Noelle Rohatinsky, Payam Dehghani, J. Patrick Neary

**Affiliations:** † University of Saskatchewan, Saskatoon, Saskatchewan, Canada; ‡ University of Regina, Regina, Saskatchewan, Canada

**Keywords:** cannabis, education, quantitative research, sports medicine, stigma

## Abstract

**Background:**

Cannabis use among elite contact sport athletes increases with legal reform and commercial availability. Limited education in cannabinoid pharmacology, use, and risk persists among athletes and medical staff, fostering stigma and reducing evidence-based guidance and communication.

**Hypothesis:**

Athletes use cannabis but perceive stigma and discomfort discussing cannabis with medical staff.

**Study Design:**

Quantitative, descriptive cross-sectional surveys.

**Level of Evidence:**

Level 4.

**Methods:**

Two anonymous online surveys were administered to elite and professional contact sport athletes (Canada, United States, Australia) and medical and training staff (February 2023 to July 2024). Descriptive statistics assessed demographics, use patterns, knowledge, education needs, attitudes, stigma, communication, and league policies.

**Results:**

A total of 166 athletes (89.7% male, 35.2% aged 18-23 years) and 64 medical staff (physicians, athletic therapists, strength and conditioning coaches, etc) responded. Knowledge (pharmacology, interactions, dosing) was low. Overall, 56% of athletes currently used cannabinoids (81% having ever used), but only 21.7% knew the endocannabinoid system and 67.4% were unfamiliar with therapeutic dosing. Among medical staff, 59.5% were unfamiliar with mechanisms, only 14% were familiar with dosing protocols, and 71.4% lacked confidence in making treatment plans. Of the athletes, 86.5% believed cannabis had therapeutic value, 72.3% wanted education, and 74.1% favored qualified scientists as instructors. Of the medical staff, 79.5% reported strong educational need, with 100% of staff desiring athlete-specific cannabinoid education. Stigma and communication barriers were substantial: 81.3% of athletes perceived public stigma, 66.1% perceived staff stigma, and only 21.4% felt comfortable discussing cannabis with medical staff. Staff believed athletes would be honest if approached; however, <50% believed athletes trusted them enough to start the conversation. Both athletes and medical staff categorized cannabinoids as supplements or medications (unlike street drugs) and both supported legalization and a revision and update on league cannabis policies.

**Conclusion:**

Athletes and medical staff exhibit parallel knowledge gaps and strong educational needs amid high interest, use, and significant communication barriers. Evidence-informed education and stigma-reducing strategies are urgently needed.

**Clinical Relevance:**

Athletes increasingly use cannabinoids for pain, sleep, and recovery, while medical staff report insufficient training and low confidence. System-level education, communication, and policy updates may improve safety, decision-making, and trust.

Cannabis and cannabinoid use in elite and professional sports has become increasingly prominent due to evolving legal frameworks, cultural normalization, and commercial expansion of cannabinoid products marketed for pain, inflammation, and sleep.^28-30^ Notably, the World Anti-Doping Agency (WADA) removed the cannabinoid cannabidiol (CBD) from its Prohibited List in 2017 (effective January 2018), a decision that has influenced significantly the landscape of cannabinoid research and use in competitive athletics. Athletes in contact sports experience high burdens of acute and chronic pain, inflammation, and sleep disturbance, which may drive interest in cannabis-based therapeutics as alternatives or adjuncts to conventional medications.^20,29,49^ Parallel to these trends, sports medicine practitioners are encountering more questions about cannabinoids in clinical encounters, yet educational and regulatory structures have been slow to adapt.

The *Cannabis sativa* plant contains >120 phytocannabinoids, of which Δ^9^-tetrahydrocannabinol (THC) and CBD have been studied most extensively.^9,25,57^ These compounds act primarily via the endocannabinoid system, including CB_1_ and CB_2_ receptors and related signaling pathways that modulate pain, inflammation, mood, sleep, and other physiological domains.^15,57,67^ Emerging clinical and translational data suggest potential therapeutic roles for cannabinoids in chronic pain, spasticity, sleep disturbance, and anxiety, with growing but still incomplete evidence in athletic populations.^2,4,18,19,56^ At the same time, concerns persist about adverse cognitive, cardiovascular, and psychiatric effects, as well as potential performance impairment and dependency.^23,34,42,58^

In elite and professional sport, these scientific and clinical uncertainties intersect with league-level anti-doping rules, varying national and subnational legal regimes, and historical framing of cannabis as an illicit “drug of abuse.”^12,16,44,45^ This context shapes both athlete behavior and medical staff practice. Previous research in nonathlete health professions has consistently shown that physicians, pharmacists, nurses, and other clinicians feel underprepared to counsel on medical cannabis, report minimal formal education, and express a desire for structured training.^15,19,41,53^ Preliminary literature in sports settings suggests similar patterns among athletic trainers and sport physicians, as well as substantial stigma around disclosure of use by athletes.^11,21^

There is limited quantitative evidence that simultaneously characterizes knowledge, attitudes, and practices related to cannabinoids among both athletes and the medical and/or training personnel who care for them in the elite contact-sport ecosystem. Understanding both perspectives is essential for designing effective education, communication strategies, and policies that reduce harm and optimize care.

This study therefore aimed to (1) describe the cannabis use patterns, knowledge, beliefs, and educational needs regarding cannabis and cannabinoids among elite and professional contact sport athletes and their medical/training staff; (2) characterize perceived stigma and communication patterns around cannabis in sport; and (3) identify convergent recommendations for education and policy to support safer, evidence-informed cannabinoid use in elite sport.

## Methods

The study protocol, including both surveys, was approved by the University of Saskatchewan Behavioral Research Ethics Board (Application ID 3889).

This work comprises 2 descriptive, cross-sectional quantitative surveys administered to separate but related cohorts: elite and professional contact sport athletes, and the medical and training staff working with elite and professional contact sport athletes. Both surveys were anonymous and administered online via SurveyMonkey. Athlete survey data were collected over a 22-month period (February 2023 to December 2024). Medical staff survey data were collected over an 18-month period (February 2023 to July 2024). Both surveys were hosted on SurveyMonkey through a university account, with servers located in Canada and the “anonymous responses” feature activated so Internet Protocol addresses were not stored.

Recruitment for both cohorts followed parallel strategies. Study information and survey links were distributed via email, text, and social media through league and sport networks, team points-of-contact, and collaborative professional relationships. The research team did not access identifiable contact details for athletes or staff. Team-designated liaisons forwarded invitations and posters voluntarily to eligible participants. Snowball sampling was encouraged and executed, with participants invited (but not obliged) to share study materials with peers. Because the survey was distributed through peer networks and social media platforms, the total number of people who viewed the recruitment material could not be tracked, and thus a total response rate could not be calculated. For both surveys, “elite or professional contact sport” was defined as university and/or college level or professional organized sport in which physical contact (e.g., tackling, body checking, grappling, blocking, intentional collisions) is a routine and integral part of game play, with elevated risk of bodily injury, including concussion.

### Athlete Cohort

Eligible participants were current or former contact sport athletes who had played at the professional or top university level (e.g., NCAA, USports) for >30 days. Athletes from Canada, the United States (US), Australia, and selected European contexts were targeted. Exclusion criteria were failure to meet these role or level thresholds.

### Medical Staff Cohort

Eligible participants were currently employed in a medical or training staff capacity with professional, university, or junior contact sport teams, or working independently with professional or elite athletes in a licensed health-related designation. Roles included physicians, athletic therapists, physical therapists, strength and conditioning staff, and allied professionals such as dentist and nutritionist. Anyone not formally part of team medical or training staff and not working directly with elite and/or professional athletes was excluded.

No validated instruments existed that specifically captured cannabinoid-related knowledge, attitudes, and behaviors in elite contact sport environments; therefore, the research team developed 2 new surveys tailored to either athletes or medical staff, respectively. Both instruments were informed by previous surveys of health professionals and general populations regarding medical cannabis education and practice, as well as published athlete-focused cannabis surveys.^8,41,47,61^

Content domains for both surveys included:

- demographics and sport/role characteristics;
- general knowledge and familiarity with cannabis, cannabinoids, and laws;
- perceptions of therapeutic benefits and risks;
- stigma and comfort discussing cannabis in the sport context;
- communication patterns between athletes and medical staff;
- educational experiences and perceived needs;
- personal cannabis and cannabinoid product use (athletes); and
- clinical experience treating athletes with cannabinoids (medical staff).

The athlete survey was piloted with 5 noncontact sport athletes to assess clarity, relevance, and length. The medical staff survey was piloted with 4 nonathlete medical professionals. Based on feedback, wording and response options were refined for readability and face validity.

Before accessing survey items, all participants viewed online information about study aims, risks, and voluntary participation and were required to select an “agree” button to indicate consent. Surveys required approximately 10 to 15 minutes to complete. Participants were free to skip questions; therefore, denominators vary by item.

Items used Likert-type response scales (e.g., strongly agree to strongly disagree; very familiar to not at all familiar) or categorical options (e.g., role, league, country, use history). For both cohorts, descriptive statistics (sample size n and proportion percent) were used to summarize responses. All analyses were conducted in Microsoft Excel (Version 2021, Microsoft 365; Microsoft Corp).

## Results

### Participant Characteristics

The athlete cohort comprised 166 respondents. Respondents were predominantly male (89.7%) and aged 18 years to 23 years (35.2%), with a secondary concentration in the 30- to 35-year-old range (22.1%). Primary league affiliations included University Sports (USports) (26.4%), National Collegiate Athletic Association (NCAA) (18.1%), National Football League (NFL) (16%), and National Hockey League (NHL) (9%), with additional representation from other professional and university leagues (Supplementary Figure 1a, available in the online version of this article). Geographically, 51% of athletes were from Canada, 47% from the US, and a small minority from Australia or Europe. Approximately 43.1% were current athletes and 56.9% retired, with most (55.2%) having played professionally or at the college level for 2 to 5 years (Supplementary Figure 1b, available online).

The medical staff cohort included 64 respondents. Among those reporting demographics, the largest age groups were 30 to 35 years and 41 to 44 years (each 22.5%) (Supplementary Figure 10a, available online). Most had 6 to 15 years of practice experience (42.6%) (Supplementary Figure 10b, available online). The majority were male (57.1%). Common professional roles (n = 50) included physicians (29.7%), athletic therapists (27.0%), and strength and conditioning staff (29.7%), with physical therapists, other training staff, and dentists comprising the remainder (Supplementary Figure 11a, available online). In terms of affiliation (n = 43), 32.6% worked with an NHL team, and 39.5% worked independently with professional athletes across sports and the remainder worked in a variety of other college and professional leagues. Most practiced in the US (65.2%), followed by Canada (32.6%), with smaller numbers in other regions (Supplementary Figure 11b, available online).

### Substance Use and Cannabis Exposure Among Athletes

A large proportion of athletes regularly used dietary supplements such as protein powders and preworkout formulations (69.4% of 144 respondents) (Supplementary Figure 2a, available online). In contrast, 66.7% reported not regularly taking medications, including common over-the-counter analgesics (Supplementary Figure 2b, available online). Alcohol use was common, with 73.6% indicating some level of recreational alcohol consumption, with only 26.4% reporting complete abstinence (Supplementary Figure 2c, available online). Tobacco use was less prevalent: 68.1% reported no regular use, 16.7% used tobacco regularly, and 15.3% used it occasionally (Supplementary Figure 2d, available online).

Cannabis and cannabinoid use were widespread. Among 105 respondents, 81% reported lifetime use of cannabis or phytocannabinoid products at one time or another (Supplementary Figure 9a, available online), with 56% currently using cannabis or cannabinoid products (Supplementary Figure 9b, available online). Product choices and doses were variable and made personally by each individual athlete, none were prescribed or recommended by team medical staff. When asked about the primary intent of their use, 38.1% described it as recreational, 35.2% as both medical and recreational, and 8.6% as medical only (Supplementary Figure 9c, available online). When asked why they take cannabinoid products and what issues they were looking to address, 44.6% utilize it with the intent to decrease pain, 42.5% are looking to improve sleep, 34.9% were aiming to enhance recovery time, and 35.9% are looking to address mental health issues. When asked what they primarily consume, most reported using both THC and CBD (38.7%), followed by THC only (22.6%), followed by CBD only (8.5%) (Supplementary Figure 9d, available online). Routes of administration were diverse, with many athletes using multiple forms: 54.3% smoked in a joint or pipe, 52.4% consumed edibles, 25.7% used vaporizers, and 20% to 25% used oils, topicals, or tinctures, with 23.8% of surveyed athletes not using cannabis in any form (Supplementary Figure 9e, available online).

Reported positive effects resulting from cannabis and cannabinoid use were common. Among 102 respondents, 63.7% reported improved sleep, 55.9% relaxation, 50% reduced pain and inflammation, 43.1% decreased anxiety, 32.4% faster recovery, 26% improved athletic performance, 25.5% reduced muscle spasms, and 22.6% increased energy (Supplementary Figure 9f, available online). Negative effects were less frequent, with 56.9% reporting no adverse effects at all; however, notable negative effects included 24.5% reported anxiety or paranoia, 26.5% increased appetite, and only 2% worse athletic performance.

### Cannabis-Related Knowledge and Familiarity

General familiarity with therapeutic uses and risks of cannabis was moderate in athletes, with only 10% of athletes reported being entirely unfamiliar with any risks or benefits. Familiarity with local laws and regulations was substantial, with 72% citing familiarity with local rules and directives. However, specific pharmacological knowledge was limited, with only 21.7% familiar with the ways cannabinoids work via the endocannabinoid system (Supplementary Figure 3a, available online). Over half (56.6%) reported little to no familiarity with the effects of individual phytocannabinoids such as THC, CBD, and CBG (Supplementary Figure 3b, available online). Awareness of drug-drug interactions was particularly low, with only 7% citing familiarity (Supplementary Figure 3c, available online). Familiarity with administration routes was much higher, with 66.7% at least somewhat familiar with nonsmoked alternative routes such as edibles, oils, and topicals (Supplementary Figure 3d, available online).

Knowledge about optimal dosing and therapeutic protocols was limited in athletes. Overall, 67.4% of athletes were not familiar with dosing requirements, optimal dosing strategies or how to structure cannabinoid use over the day or season (Supplementary Figure 3e, available online). Most athletes believed at least a “moderate amount” of research existed on both harms (72.9%) and benefits (81.4%) of medical cannabis, but this did not translate into confidence in their own understanding.

Medical staff reported a similar pattern of partial awareness but significant gaps in detailed knowledge. Over half indicated some familiarity with therapeutic uses and risks, yet 59.5% were not familiar with the unique pharmacological effects of individual phytocannabinoids and the same proportion were not familiar with molecular mechanisms of action via the endocannabinoid system (Supplementary Figure 12a, available online). Most medical staff did not know how cannabinoids worked in the human body. Only 11.9% felt familiar with drug-drug interactions involving cannabinoids with 61.9% not at all or not so familiar (Supplementary Figure 12b, available online).

Familiarity with recreational or therapeutic dosing was low in medical staff. Only 14% reported being very or extremely familiar with dosing protocols and strategies for structuring cannabinoid use (Supplementary Figure 13a, available online). This translated into low confidence in clinical practice, with 71.4% reporting having no confidence in designing treatment plans (including dosing) for athletes interested in cannabinoid use (Supplementary Figure 13b, available online).

### Attitudes Toward Therapeutic and Recreational Use

Athletes generally held positive, or at least accepting, attitudes toward both medical and recreational cannabis use. Among 111 respondents, 86.5% agreed or strongly agreed that cannabis has medical benefits (Supplementary Figure 5a, available online), and only 8% held the view that there are no medicinal benefits and people “simply want to get high” (Supplementary Figure 5b, available online). A large majority (81.1%) believed therapeutic use should undergo a risk-benefit analysis similar to other medications. Regarding acceptability, 81.3% viewed medical use of cannabis or phytocannabinoids as acceptable, with 0% finding it totally unacceptable (Supplementary Figure 5c, available online). For recreational use, 70.5% considered it acceptable to some degree, while only 6.3% found it completely unacceptable (Supplementary Figure 5d, available online). Only about one-fifth (20.5%) expressed no interest in using cannabis for any reason.

Concerns about performance impairment during the season were relatively low; only 25% of athletes agreed that fear of impaired performance deterred them from using cannabis (Supplementary Figure 5e, available online). In contrast, substitution motives were prominent: 58.6% would consider using cannabis during the season as a substitute for recreational alcohol, tobacco, or other drugs if allowed (Supplementary Figure 5f, available online), and 67% would consider therapeutic use as a substitute for other prescription or over-the-counter drugs if effective and permitted (Supplementary Figure 7a, available online). Many athletes (63.4%) wished their league allowed recreational or therapeutic cannabis use without punishment or testing (Supplementary Figure 7b, available online).

Medical staff expressed largely supportive attitudes toward cannabis legalization and therapeutic use. Nearly all (97.4%) supported legalization, and 44.7% reported personal use of cannabis or phytocannabinoids either recreationally or therapeutically. When asked how they categorize cannabinoids, 61.1% aligned them with prescription medications (good safety profile, effective when used with clinician oversight), and 41.7% aligned them with over-the-counter medications (good safety profile, low harm, self-directed dosing possible). Only 19.4% categorized cannabis like alcohol, and none categorized it as a dangerous street drug with high harm potential (Supplementary Figure 17a, available online). A substantial majority (71.8%) wanted their league policies reviewed, updated and clarified regarding both therapeutic and recreational cannabis use (Supplementary Figure 17b, available online).

Athletes similarly tended to categorize cannabis in medical rather than illicit terms. About 74.1% categorized it as medicinally useful similar to prescription medications for anxiety, depression, or sleep (Supplementary Figure 6a, available online), and 55.4% likened it to over-the-counter pain relievers (Supplementary Figure 6b). While 44.6% categorized it like alcohol for recreational use (Supplementary Figure 6c, available online), only 10.7% grouped it with hard street drugs such as cocaine.

### Stigma, Trust, and Communication

Stigma and communication barriers were central themes, particularly from the athlete perspective. Among 112 athlete respondents, 81.3% believed the public holds negative stigma toward cannabis use in athletics, 66.1% perceived negative stigma from medical staff, trainers, and management, and 40.2% believed other athletes stigmatize cannabis discussions or use. A majority (68.5%) felt they would be stigmatized for talking about or asking about cannabis with their team trainers and doctors (Supplementary Figure 4a, available online), and 62.2% believed team doctors and trainers might find it uncomfortable to be asked about cannabis (Supplementary Figure 4b, available online). Only 21.4% agreed that they felt comfortable discussing cannabis with team medical staff (Supplementary Figure 4c, available online). Reflecting this, 80.4% reported never having discussed cannabis with team trainers or doctors (Supplementary Figure 4d, available online). When asked about disclosure, only 41.1% said they would tell team medical staff about cannabis use, while 60.1% indicated they would keep use a secret (Supplementary Figure 4e, available online).

Medical staff responses suggested partial awareness of this relational tension. Approximately two-thirds believed athletes would be honest if asked directly about cannabis use, but only 43% believed athletes trusted them enough to seek information about cannabinoid products. Nearly two-thirds (64.1%) had noticed increasing interest from players in cannabis and cannabinoid products as therapeutic options (Supplementary Figure 16, available online). Conversations about cannabis were more often initiated by athletes than by staff; in the medical staff sample, 48.7% of cannabis discussions were athlete-initiated, while only 10.3% were initiated by staff themselves.

### Education, Confidence, and Perceived Needs

Among athletes, 72.3% expressed interest in learning more about the therapeutic uses of cannabis and phytocannabinoids. A substantial majority (74.1%) stated they would like to learn about cannabinoid mechanisms and therapeutic applications from qualified cannabis scientists (Supplementary Figure 8a, available online). Many (61.2%) indicated they would feel more comfortable discussing therapeutic cannabinoid use with medical staff if they themselves had more education on the topic (Supplementary Figure 8b, available online). Only 11.6% believed that team doctors and trainers were sufficiently educated and well equipped to answer their questions about recreational or therapeutic cannabis use (Supplementary Figure 8c, available online). In contrast, 80.4% reported they would feel more comfortable if physicians and trainers recommending cannabinoids were required to complete specific training or licensing (Supplementary Figure 8d, available online).

Medical staff reported very limited formal education on cannabis during their professional training, with most describing zero dedicated hours in the medical curriculum completed to attain their designation and license. Consistent with their perceived deficits, 74.4% of medical staff reported not feeling sufficiently educated to discuss cannabis use of any kind with athletes (Supplementary Figure 15c, available online). Only a small minority had treated an athlete using cannabis or phytocannabinoid medicine in the role of a cannabis clinician (Supplementary Figure 14b, available online). Nevertheless, motivation to learn was high: 87.2% were interested in learning more about therapeutic uses of cannabinoids (Supplementary Figure 15a, available online), and 79.5% felt they needed education on cannabis and phytocannabinoids in medicine (Supplementary Figure 14a, available online). 100% of respondents expressed interest in a peer-reviewed education package designed specifically for the athlete population and taught by qualified educational instructors, if one existed (Supplementary Figure 15b, available online).

## Discussion

This combined analysis of elite contact sport athletes and their medical and training staff reveals parallel and mutually reinforcing gaps in cannabinoid knowledge, confidence, and communication in high-performance sport environments. Athletes perceive substantial therapeutic potential and report high levels of lifetime and current cannabis and cannabinoid product use, yet demonstrate limited understanding of underlying pharmacology, dosing, and interactions. Medical staff, who are tasked with protecting athlete health and safety, similarly report limited education, low confidence, and a strong desire for further training.

### Converging Knowledge Gaps and Educational Demand

The findings align with previous research in broader clinical populations that has documented insufficient coverage of medical cannabis in undergraduate and postgraduate curricula,^5^ and low provider confidence in dosing, indications, and risk mitigation.^22,50,62,65,66^ In sports medicine, the present data extend this literature by quantifying similar deficits among physicians, athletic therapists, and strength and conditioning professionals working directly with elite athletes.^31,33,37,56^ Despite increasing athlete queries and personal support for legalization, most medical staff reported being unfamiliar with core concepts such as the endocannabinoid system, specific phytocannabinoid profiles, and drug-drug interactions, with nearly three-quarters having no confidence in designing cannabinoid treatment plans, which aligns with research done in other demographics.^3,50,54,62^

Athletes, meanwhile, reported high interest in therapeutic applications for pain, sleep, and recovery, but similarly lacked detailed knowledge of cannabinoid science.^29,40,49,63^ The mismatch between perceived benefits and limited understanding raises concerns about self-directed experimentation without evidence-based guidance, particularly in a commercial environment dominated by aggressive marketing and variable product quality.^23,29,30,32^ The very high uptake of cannabinoids among respondents suggests that current educational and regulatory structures are lagging behind real-world behavior.^7,29,49,55,63^

Importantly, both groups expressed strong and specific educational desires: athletes wanted to learn from qualified cannabis scientists and wanted medical staff to complete formal training, while medical staff wanted peer-reviewed, athlete-specific education packages, similar to what has been found when investigating other demographics.^29,40,48,59^ This bidirectional demand suggests a receptive context for structured curricula, continuing professional development programs, and integrated athlete education modules.^17,27,48,63,64^

### Stigma, Secrecy, and Fractured Communication

Perhaps the most striking divergence between athletes and medical staff lies not in fundamental attitudes toward cannabis (both cohorts were broadly supportive of medical use and legalization) but in their relational experience of stigma and trust. Supporting previous research, athletes reported widespread perceived stigma from the public, medical staff, and management, and described feeling that discussion of cannabis with team doctors and trainers would be stigmatizing and uncomfortable.^51,56,63^ Most had never broached the topic with medical staff, and a majority indicated they would conceal cannabis use.

From a clinical risk-management perspective, this secrecy is problematic. If athletes are using cannabinoids to manage pain, sleep, or mental health, but are not disclosing this to medical staff, critical aspects of their pharmacologic profile and symptom burden may be missing from clinical assessments.^7,30,59^ Potential drug-drug interactions, adverse effects, and opportunities for safer doses or ratio alternatives may go undetected.^43,46,59^ The findings mirror previous work on stigma as a barrier to help-seeking in mental health and substance use among athletes, suggesting that cannabis has become another domain where silence and mistrust undermine comprehensive care.^10,24,26,38,52^

Medical staff, for their part, believed that many athletes would be honest if asked directly, but acknowledged that relatively few athletes sought them out for cannabinoid guidance.^33,39^ Low confidence and low education may contribute to avoidance behavior among staff, which athletes may interpret as disinterest or disapproval, further entrenching stigma and secrecy.^13,33,39,48^ Recently published qualitative work by the authors suggests that some medical staff deliberately avoid cannabis conversations because they feel unable to offer evidence-based advice, inadvertently reinforcing athlete perceptions that the topic is off-limits.^36,39,48^

### Policy and Categorization: an Opportunity for Alignment

Both cohorts showed a consistent pattern of categorizing cannabinoids closer to legitimate therapeutic agents than to illicit drugs, which is supported by past research publications.^33,48,60,63^ Athletes most often likened cannabis to supplements, prescriptions, or over-the-counter medications. Medical staff similarly situated cannabis alongside prescription and nonprescription medications with good safety profiles and potential medical value, and none categorized it as a dangerous street drug. These categorizations are congruent with evolving scientific views of cannabinoids as pharmacologically active agents with both potential benefits and risks, rather than purely recreational substances.^27,30,37,56^

Coupled with broad support for legalization and strong interest in updated league policies, this reframing creates an opportunity for coherent, medically grounded policy reform in elite sport.^1^ Many athletes expressed a desire for league regulations that would permit therapeutic and even recreational cannabis use under clear guidelines, while medical staff sought review and modernization of league rules in light of changing societal attitudes and emerging evidence. Harmonizing sport policies with contemporary science and public health principles could reduce stigma, support more transparent athlete-provider communication, and provide a framework for safe,^6^ monitored use where appropriate.^1,14,23,27,35,56^

### Implications for Education, Practice, and Research

The combined data from athletes and medical staff suggest several high-priority directions:

1. Integrated education across stakeholders: comprehensive, peer-reviewed education on cannabinoid pharmacology, indications, contraindications, and dosing should be developed for both medical staff and athletes, with content tailored to their distinct roles and baseline knowledge. For medical staff, this might include accredited continuing education modules and formal certification pathways. For athletes, education can be embedded into existing team seminars on nutrition or mental health, emphasizing harm reduction, realistic expectations, and communication with qualified clinicians.
2. Structured communication channels: teams and leagues should create explicit, confidential avenues for athletes to discuss cannabis use and questions with medical staff without fear of punitive consequences. Training in communication skills and stigma reduction, drawing on models from mental health and anti-doping education, may help medical staff invite disclosure and respond nonjudgmentally while still addressing safety and rule compliance.
3. Evidence-informed guidelines: multidisciplinary expert groups in sports medicine, pharmacology, and policy should develop consensus guidance on potential therapeutic cannabinoid use in sport, including indications (e.g., chronic pain, sleep disturbance), contraindications (e.g., certain psychiatric or cardiovascular conditions), monitoring, and interactions with existing anti-doping rules. Clear, accessible guidance may reduce reliance on nonexpert sources and commercial marketing.
4. Further research: future work should use larger, more diverse cohorts across sports, sexes, and regions, and employ robust designs including longitudinal and interventional studies to assess the impact of cannabinoid education, policy changes, and clinical protocols on knowledge, behavior, health outcomes, and performance.

## Limitations

This integrated analysis shares the limitations of the 2 underlying surveys. The cross-sectional, self-selected samples limit causal inference and generalizability. Cross-sectional designs preclude temporal or causal conclusions regarding the effects of education, policy, or cannabis use on health and performance. The custom-made survey instruments, while content-validated via piloting, lack formal psychometric validation, which may affect reliability and comparability. Self-report is vulnerable to social desirability bias, particularly given legal and policy sensitivities around cannabis, which may lead to under- or over-reporting of use and stigma.

Sampling could have been subjected to selection bias where people with greater interest in cannabis may have been more inclined to participate, potentially inflating prevalence estimates of use, interest in education and a potential bias towards positive effects of cannabis products. The athlete sample, while spanning multiple leagues and countries, may not represent all contact sport athletes, and the medical staff cohort was relatively small. With snowball sampling, researchers could not control the final distribution or uptake of the survey link nor calculate a response rate. Finally, neither survey stratified results by sport, position, or specific league rules, which could influence both behavior and attitudes

## Conclusion

Elite contact sport athletes and their medical and training staff are navigating a rapidly changing landscape of cannabis legality, commercialization, and evolving scientific evidence with limited formal education and significant uncertainty. Athletes report relatively high levels of use and strong belief in therapeutic benefits but lack detailed knowledge and experience substantial stigma and communication barriers with team medical staff. Medical staff, while largely supportive of legalization and open to therapeutic possibilities, frequently feel undereducated and lack confidence in providing cannabinoid guidance.

This misalignment creates risk: athletes may self-experiment with potent, commercially driven products without adequate clinical oversight, while medical professionals, constrained by educational and policy gaps, may avoid conversations that could enhance safety and trust. Addressing these issues requires coordinated educational, clinical, and policy responses that involve both athletes and medical staff, reframing cannabinoids from taboo substances to complex therapeutic tools that demand rigorous, evidence-based management in the high-stakes context of elite contact sport.

## Supplemental Material

sj-docx-1-sph-10.1177_19417381261495512 – Supplemental material for Athletes and Medical Staff in Elite Contact Sports: a Quantitative Analysis of Use, Knowledge, and Attitudes Regarding Cannabinoids Supplemental material, sj-docx-1-sph-10.1177_19417381261495512 for Athletes and Medical Staff in Elite Contact Sports: a Quantitative Analysis of Use, Knowledge, and Attitudes Regarding Cannabinoids by Elizabeth S. Thompson, Jane Alcorn, Robert Laprairie, Noelle Rohatinsky, Payam Dehghani and J. Patrick Neary in Sports Health
