# Supplementary material for: Athletes and Medical Staff in Elite Contact Sports: a Quantitative Analysis of Use, Knowledge, and Attitudes Regarding Cannabinoids
Source: Sports Health. 2026 Oct 21:19417381261495512. Online ahead of print. doi: 10.1177/19417381261495512 (PMC13687462; doi:10.1177/19417381261495512)
Supplement: Supplementary file 1 [file sj-docx-1-sph-10.1177_19417381261495512.docx]

**Supplementary Figures**

**Athlete Data**

**Fig.1 a)** League Affiliation (n=144)

**b)** How many years have you (did you) play professionally and/or college level? (n=143)

|  | Responses |
| --- | --- |
| USports | 26.39% |
| NCAA | 18.06% |
| NFL | 15.97% |
| Other | 15.97% |
| NHL | 9.03% |
| CHL | 6.94% |
| AHL | 5.56% |
| CFL | 2.08% |
| UFC | 0% |
| MLB | 0% |

|  | Responses |
| --- | --- |
| <2 | 15.38% |
| 2-5 | 55.24% |
| 6-10 | 18.88% |
| 11-15 | 6.99% |
| 16+ | 3.5% |

|  | Responses |
| --- | --- |
| Yes | 69.44% |
| No | 30.56% |

|  | Responses |
| --- | --- |
| Yes | 33.33% |
| No | 66.67% |

b) Do you regularly take medications of any kind? (including over the counter such as Aleve or Tylenol) (n=144)

**Fig. 2 a)** Do you regularly take supplements of any kind? (including protein powder, pre-workout, creatine, etc.) (n=144)

|  | Responses |
| --- | --- |
| Yes | 27.08% |
| No | 26.39% |
| Only occasionally | 46.53% |

|  | Responses |
| --- | --- |
| Yes | 16.67% |
| No | 68.06% |
| Only occasionally | 15.28% |

d) Do you regularly smoke or chew tobacco (n=144)

**c)** Do you regularly consume alcohol recreationally? (n= 144)

b) How familiar are you with the unique effects of varying phytocannabinoids, i.e. THC, CBD, CBG, CBN, etc. (n= 129)

**Fig. 3 a)** How familiar are you with the ways phytocannabinoids work within cells (i.e., the endocannabinoid system)? (n=129)

|  | Responses |
| --- | --- |
| Extremely familiar | 3.1% |
| Very familiar | 5.43% |
| Somewhat familiar | 13.18% |
| Not so familiar | 35.66% |
| Not at all familiar | 42.64% |

|  | Responses |
| --- | --- |
| Extremely familiar | 4.65% |
| Very familiar | 8.53% |
| Somewhat familiar | 30.23% |
| Not so familiar | 27.91% |
| Not at all familiar | 28.68% |

**d)** How familiar are you with alternative (i.e., non-smoked) routes of administration of cannabis? (n=129)

**c)** How familiar are you with drug-drug interactions involving phytocannabinoids and other prescription and non-prescription medications? (n=129)

|  | Responses |
| --- | --- |
| Extremely familiar | 2.33% |
| Very familiar | 4.65% |
| Somewhat familiar | 21.71% |
| Not so familiar | 35.66% |
| Not at all familiar | 35.66% |

|  | Responses |
| --- | --- |
| Extremely familiar | 16.28% |
| Very familiar | 19.38% |
| Somewhat familiar | 31.01% |
| Not so familiar | 23.26% |
| Not at all familiar | 10.08% |

e) How familiar are you with the dosage required for therapeutic purposes and how best to structure use protocols throughout the day/night and over the course of time? (n= 129)

|  | Responses |
| --- | --- |
| Extremely familiar | 5.43% |
| Very familiar | 6.98% |
| Somewhat familiar | 20.16% |
| Not so familiar | 36.43% |
| Not at all familiar | 31.01% |

**b)** Team doctors and trainers might find it uncomfortable to be asked about cannabis (n=111)

**Fig.4 a)** I feel/felt I would be stigmatized talking or asking about cannabis with my team trainers and doctors (n = 111)

|  | Responses |
| --- | --- |
| Strongly agree | 24.32% |
| Agree | 44.14% |
| Neither agree nor disagree | 20.72% |
| Disagree | 8.11% |
| Strongly disagree | 2.7% |

|  | Responses |
| --- | --- |
| Strongly agree | 14.41% |
| Agree | 47.75% |
| Neither agree nor disagree | 27.93% |
| Disagree | 9.01% |
| Strongly disagree | 0.9% |

**d)** I have *never* discussed cannabis with my team trainers and doctors (n=112)

c) I feel/felt comfortable discussing cannabis with my team trainers and doctors (n= 112)

|  | Responses |
| --- | --- |
| Not Sure | 4.46% |
| Strongly agree | 4.46% |
| Agree | 16.96% |
| Neither agree nor disagree | 25% |
| Disagree | 33.93% |
| Strongly disagree | 15.18% |

|  | Responses |
| --- | --- |
| True | 80.36% |
| False | 19.64% |

**e)** If I use/used cannabis (therapeutically or recreationally) I would keep it secret from medical staff. (n=111)

|  | Responses |
| --- | --- |
| Yes | 60.36% |
| No | 39.64% |

**Fig.5 a)** I believe that cannabis has medical benefits (n= 111)

b) I do not believe there are medicinal benefits to cannabis. People just want to get high. (n= 112)

|  | Responses |
| --- | --- |
| Strongly agree | 42.34% |
| Agree | 44.14% |
| Neither agree nor disagree | 9.91% |
| Disagree | 3.6% |
| Strongly disagree | 0% |

|  | Responses |
| --- | --- |
| Strongly agree | 2.68% |
| Agree | 5.36% |
| Neither agree nor disagree | 15.18% |
| Disagree | 33.04% |
| Strongly disagree | 43.75% |

|  | Responses |
| --- | --- |
| Completely acceptable | 60.71% |
| Somewhat acceptable | 20.54% |
| Neutral | 16.07% |
| Somewhat unacceptable | 2.68% |
| Completely unacceptable | 0% |

|  | Responses |
| --- | --- |
| Completely acceptable | 49.11% |
| Somewhat acceptable | 21.43% |
| Neutral | 19.64% |
| Somewhat unacceptable | 3.57% |
| Completely unacceptable | 6.25% |

d) In general, I believe that *recreational* use of cannabis or phytocannabinoids is (n=112)

**c)** In general, I believe that *medical/therapeutic* use of cannabis or phytocannabinoids is (n= 112)

|  | Responses |
| --- | --- |
| Strongly agree | 13.39% |
| Agree | 11.61% |
| Neither agree nor disagree | 21.43% |
| Disagree | 30.36% |
| Strongly disagree | 23.21% |

|  | Responses |
| --- | --- |
| Strongly agree | 26.13% |
| Agree | 32.43% |
| Neither agree nor disagree | 12.61% |
| Disagree | 18.02% |
| Strongly disagree | 10.81% |

**e)** I would not/did not use cannabis during the athletic season because I think it could impair my performance. (n=112)

**f)** I would/did consider using cannabis during the athletic season as a substitute for recreational alcohol/tobacco/other drugs (if it was allowed). (n=111)

c) I categorize cannabis in the same group as alcohol (i.e., for entertainment/recreational purposes) (n=112)

**Fig.6** a) I categorize cannabis as medicinally useful similar to prescription drugs for anxiety, depression, or sleep troubles (n=112)

b) I categorize cannabis as medicinally useful similar to prescription or over-the-counter pain reduction medications (i.e., Aleve, Tylenol) (n=112)

|  | Responses |
| --- | --- |
| Strongly agree | 25.89% |
| Agree | 48.21% |
| Neither agree nor disagree | 13.39% |
| Disagree | 9.82% |
| Strongly disagree | 2.68% |

|  | Responses |
| --- | --- |
| Strongly agree | 16.96% |
| Agree | 38.39% |
| Neither agree nor disagree | 23.21% |
| Disagree | 17.86% |
| Strongly disagree | 3.57% |

|  | Responses |
| --- | --- |
| Strongly agree | 16.07% |
| Agree | 28.57% |
| Neither agree nor disagree | 19.64% |
| Disagree | 23.21% |
| Strongly disagree | 12.5% |

|  | Responses |
| --- | --- |
| Strongly agree | 32.14% |
| Agree | 34.82% |
| Neither agree nor disagree | 14.29% |
| Disagree | 10.71% |
| Strongly disagree | 8.04% |

|  | Responses |
| --- | --- |
| N/A : My league allows cannabis use | 7.14% |
| Strongly agree | 31.25% |
| Agree | 32.14% |
| Neither agree nor disagree | 23.21% |
| Disagree | 4.46% |
| Strongly disagree | 1.79% |

**b)** I wish my league had regulations that allowed me to use cannabis either recreationally or therapeutically without punishment (n=112)

**Fig. 7 a)** I would/did consider using cannabis during the athletic season medically/therapeutically as a substitute for other prescription or over-the-counter drugs if it helped my medical issue (if it was allowed). (n=112)

**Fig. 8 a)** I would like to learn more about the therapeutic uses of cannabis ( including basic mechanisms of action) from qualified cannabis scientists. (n=112)

**b)** I would feel more comfortable discussing the therapeutic use of cannabis and/or phytocannabinoids with my team medical staff if I had more education about it. (n=129)

|  | Responses |
| --- | --- |
| Strongly agree | 31.25% |
| Agree | 42.86% |
| Neither agree nor disagree | 17.86% |
| Disagree | 5.36% |
| Strongly disagree | 2.68% |

|  | Responses |
| --- | --- |
| Strongly agree | 19.38% |
| Agree | 41.86% |
| Neither agree nor disagree | 32.56% |
| Disagree | 3.88% |
| Strongly disagree | 2.33% |

c) Team doctors and trainers are sufficiently educated on therapeutic cannabis uses and are well-equipped to answer my questions (n=112)

d) I would feel more comfortable if physicians/trainers who recommended cannabis or phytocannabinoids for therapeutic purposes were required to undergo a specific training or licensing program to do so (n=112)

|  | Responses |
| --- | --- |
| Strongly agree | 1.79% |
| Agree | 9.82% |
| Neither agree nor disagree | 37.5% |
| Disagree | 35.71% |
| Strongly disagree | 15.18% |

|  | Responses |
| --- | --- |
| Strongly agree | 28.57% |
| Agree | 51.79% |
| Neither agree nor disagree | 17.86% |
| Disagree | 1.79% |
| Strongly disagree | 0% |

**b)** Do you currently use cannabis or phytocannabinoids of any kind? (n=105)

**Fig. 9** a) Have you ever used cannabis or phytocannabinoids (including THC and/or CBD isolates) in the past? (n=105)

|  | Responses |
| --- | --- |
| Yes | 80.95% |
| No | 19.05% |

|  | Responses |
| --- | --- |
| Yes | 56.19% |
| No | 43.81% |

c) Was/is your cannabis/phytocannabinoid use medical, recreational, or both? (n=105)

|  | Responses |
| --- | --- |
| I do not use Cannabis | 18.1% |
| Medical | 8.57% |
| Recreational | 38.1% |
| Both Medical and Recreational | 35.24% |

d) What do you primarily use: THC, CBD, both, or other? (n=106)

|  | Responses |
| --- | --- |
| Both THC and CBD | 38.68% |
| THC | 22.64% |
| I do not use cannabis | 21.7% |
| CBD | 8.49% |
| Other (please specify) | 4.72% |
| I have no idea, I use whatever is shared with me | 3.77% |

|  | Responses |
| --- | --- |
| Smoke in joint or pipe | 54.29% |
| Edible (cookie, candy, etc.) | 52.38% |
| Vaporizer | 25.71% |
| Topical (cream, ointment, patch) | 24.76% |
| Oil | 23.81% |
| N/A I don't use cannabis in any form | 23.81% |
| Tincture | 12.38% |
| Capsule | 8.57% |
| Spray | 3.81% |
| Other (please specify) | 2.86% |

**e)** How do you use cannabis/phytocannabinoids? (check all that apply) (n=105)

**f)** Have you had any positive effects from cannabis/phytocannabinoids use? (check all that apply) (n=102)

|  | Responses |
| --- | --- |
| Helps with sleep | 63.73% |
| Calms me down/helps me relax | 55.88% |
| Less pain and inflammation | 50% |
| Euphoria( i.e., feelings of pleasure, excitement, wellbeing, happiness) | 44.12% |
| Decreased anxiety | 43.14% |
| Faster recovery | 32.35% |
| Improved athletic performance | 26.47% |
| Fewer muscle spasms | 25.49% |
| Increased energy | 22.55% |
| Decreased nausea | 19.61% |
| No, I have never had any positive effects | 16.67% |
| Other (please specify) | 7.84% |

**Medical Staff Data**

|  | Responses |
| --- | --- |
| <18 | 0% |
| 18-23 | 4.08% |
| 24-29 | 18.37% |
| 30-35 | 22.45% |
| 36-40 | 8.16% |
| 41-44 | 22.45% |
| 45-49 | 4.08% |
| 50-54 | 6.12% |
| 55-59 | 10.2% |
| 60+ | 4.08% |

|  | Responses |
| --- | --- |
| <2 | 8.51% |
| 2-5 | 17.02% |
| 6-15 | 42.55% |
| 16-25 | 17.02% |
| 26+ | 14.89% |

**Fig. 10** **a)** Age category (*n* = 49) **b)** Number of years in practice *(n = 47)*

**Fig.11** **a)** Official role on team / licensed designation (*n* = 50) **b)** Geographical Location (*n= 46*)

|  | Responses |
| --- | --- |
| Doctor | 29.73% |
| Athletic Therapist | 27.03% |
| Strength and Conditioning staff | 29.73% |
| Physio Therapist | 8.11% |
| Dietitian / Nutritionist | 0% |
| Training Staff | 2.7% |
| Dentist | 2.7% |
| Chiropracter | 0% |

|  | Responses |
| --- | --- |
| Canada | 32.61% |
| United States | 65.22% |
| Australia | 2.17% |
| Europe | 0% |

**Fig.12 a)** How familiar are you with the molecular mechanism of action of cannabis (i.e., endocannabinoid system)? (*n= 42)*

**b)** How familiar are you with drug-drug interactions involving phytocannabinoids and other prescription and non-prescription? (*n= 42)* medications?

|  | Responses |
| --- | --- |
| Extremely familiar | 11.9% |
| Very familiar | 7.14% |
| Somewhat familiar | 21.43% |
| Not so familiar | 35.71% |
| Not at all familiar | 23.81% |

|  | Responses |
| --- | --- |
| Extremely familiar | 7.14% |
| Very familiar | 4.76% |
| Somewhat familiar | 26.19% |
| Not so familiar | 28.57% |
| Not at all familiar | 33.33% |

**b)** How confident are you in creating treatment plans (e.g., dosing) for patients/athletes wanting to use cannabis or phytocannabinoids therapeutically? *(n=42)*

**Fig.13 a)** How familiar are you with the dosage required for therapeutic purposes and how best to structure usage protocols throughout the day/night and over the course of time? *(n=42)*

|  | Responses |
| --- | --- |
| Extremely familiar | 9.52% |
| Very familiar | 4.76% |
| Somewhat familiar | 14.29% |
| Not so familiar | 38.1% |
| Not at all familiar | 33.33% |

|  | Responses |
| --- | --- |
| Extremely confident | 14.29% |
| Very confident | 7.14% |
| Somewhat confident | 7.14% |
| Not so confident | 30.95% |
| Not at all confident | 40.48% |

**Fig.14 a**) How strongly do you feel that you need education on cannabis and phytocannabinoids in medicine? (*n=39)*

**b)** Have you ever treated an athlete or patient using cannabis or phytocannabinoid medicine (in the role of a cannabis clinician)? *(n= 38)*

|  | Responses |
| --- | --- |
| Strongly needed | 23.08% |
| Needed | 56.41% |
| Neutral | 17.95% |
| Unneeded | 2.56% |
| Strongly unneeded | 0% |

|  | Responses |
| --- | --- |
| Yes | 13.16% |
| No | 86.84% |

**c)** Do you feel sufficiently educated on cannabinoids to discuss cannabis use of any kind with athletes? *(n=39)*

**Fig.15 a)** I am interested in learning more about the therapeutic uses of cannabis and phytocannabinoids and how they are being used medically *(n=39)*.

**b)** If a peer-reviewed education package was created to teach therapeutic uses of cannabis specific to the athlete population, would you be interested in learning more on this topic? (*n=39)*

|  | Responses |
| --- | --- |
| Strongly agree | 46.15% |
| Agree | 41.03% |
| Neither agree nor disagree | 12.82% |
| Disagree | 0% |
| Strongly disagree | 0% |

|  | Responses |
| --- | --- |
| Yes | 100% |
| No | 0% |

|  | Responses |
| --- | --- |
| Yes | 25.64% |
| No | 74.36% |

**Fig.16** Have you noticed an increase in interest in cannabis and/or phytocannabinoids as a therapeutic option from your players? *(n=39)*

**Fig. 17 a) What category best matches how you categorize cannabis? Check all that apply *(n=36)***

**b)** I would like to see my league policies reviewed and updated with regard to cannabis use (both therapeutically and recreationally) *(n=39)*
